# From Mud to Meat: Comparative Metabarcoding Reveals Two Different Evolutionary Paths to Carnivory in a Group of Meiofaunal Annelids

**DOI:** 10.1111/mec.70151

**Published:** 2025-10-28

**Authors:** Joseph M. Mack, Alexandra E. Bely

**Affiliations:** ^1^ Department of Biology University of Maryland College Park Maryland USA

**Keywords:** annelids, carnivory, feeding, freshwater, meiofauna, metabarcoding

## Abstract

Evolutionary transitions to carnivory represent profound shifts in feeding mode that are often accompanied by widespread changes in organismal function, behaviour and ecology. Such transitions have evolved numerous times among animals, and predator–prey interactions have been major drivers of animal evolution. Despite the ecological and evolutionary importance of carnivory, the evolutionary steps leading to this feeding mode are poorly understood. Although relatively rare, lineages that have recently adopted predatory lifestyles are particularly valuable for understanding the evolution of carnivory. The annelid genus *Chaetogaster*, composed of small freshwater oligochaetes, is unusual in having recently evolved carnivory not just once but twice, making it an excellent model to infer evolutionary steps from detritivory to carnivory. We performed a gut‐content analysis of eight *Chaetogaster* species and a detritivorous outgroup, using 18S rDNA metabarcoding complemented by visual gut content assessment to infer diets. We found that species within the lineages presumed to be carnivorous had large fractions of animal metabarcoding reads, as predicted. Their closest relatives, however, differed in dietary profiles. We infer that the closest relatives of one carnivorous lineage, which are generalist predators, primarily feed on ciliates, while the closest relatives of the second carnivorous lineage, which are mollusc symbionts, are detritivores. Our data suggest that carnivory evolved two ways in *Chaetogaster*, with one transition mediated by ciliate feeding and a second mediated by symbiosis. Overall, this study suggests that carnivory can evolve from noncarnivorous ancestors through distinct evolutionary pathways, even among closely related lineages.

## 1 Introduction

Predatory interactions are among the most fundamental drivers of animal evolution. During the Cambrian, predators are thought to have stimulated evolution as predator–prey arms races drove the development of new body plans and ecosystems (Bengtson 2002). This legacy persists today, as many of the most diverse animal groups, such as birds, mammals and insects, are likely to be descended from predatory common ancestors (Román‐Palacios et al. 2019). To this day, predatory animals have powerful, well‐documented impacts on ecological communities (Beschta and Ripple 2009). Despite the importance of predation to animal ecology and evolution, little is known about how or why carnivory, defined as a diet mostly composed of animal tissue, originates in animals. This contrasts with the literature exploring carnivory among plants and fungi, where recent and repeated origins of carnivory have enabled the development of elegant cost–benefit models to explain its origins (Barron 2003; Givnish et al. 1984). Also well characterised are the molecular and ecological intermediate steps that link carnivorous plants and fungi to noncarnivorous ancestors (Liu et al. 2014; Palfalvi et al. 2020; Pavlovič and Saganová 2015; Yang et al. 2012). There is no comparable comprehensive literature for the evolution of carnivory in animals.

Our understanding of the evolution of carnivory in animal lineages is limited at least in part because recent transitions to carnivory are rare (Burin et al. 2016; Price et al. 2012; Siqueira et al. 2020). Carnivory is common across animals, but most carnivorous animals are in clades that evolved carnivory tens to hundreds of millions of years ago (e.g., mammals, arthropods, cnidarians) (Román‐Palacios et al. 2019). Of the relatively few lineages that evolved carnivory recently, only a handful have been studied. Examples include mice in the genus *Onychomys* that have shifted from omnivory to specialise on arthropod prey (Langley 1994), deep‐sea sponge species that have become crustacean predators (Vacelet and Boury‐Esnault 1995), and multiple genera of lepidopterans whose larvae have abandoned phytophagy to feed on other invertebrates (Montgomery 1983; Pierce 1995; Rubinoff and Haines 2005). Studies on these systems have revealed that the evolution of carnivory may be enabled by the development of efficient prey capture behaviours, morphological changes, preadaptive diets or symbiosis with potential prey. Despite excellent research on these study systems, there is a paucity of research comparing closely related carnivores and noncarnivores. Predatory mice, rodents and sponges consist of entire genera that are carnivorous, but groups in which carnivory evolved even more recently could prove especially useful for understanding how these transitions occur.

**FIGURE 1:**
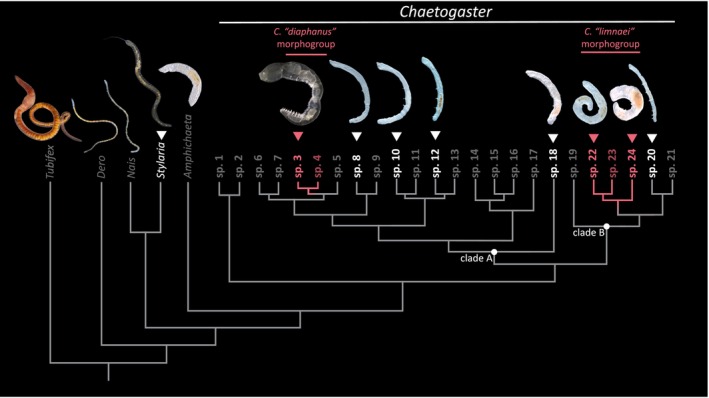
Diversity of *Chaetogaster* and naidid relatives. Species labelled in bright white or red text and with arrowheads are those represented in this study. Red branches denote the *C. ‘diaphanus’* and *C. ‘limnaei’* clades within which carnivory has evolved, while A and B represent clades that include each carnivorous lineage and its closest relatives. Relationships between *Chaetogaster* species and other naidids are based on Mack et al. (2023) and Erséus et al. (2017). Worm images have anterior ends oriented up. Images are not to scale.

Water nymph worms of the genus *Chaetogaster* (Annelida: Clitellata: Naididae: Naidinae) are a promising model to study how carnivory evolves among closely related species. This genus of freshwater, primarily asexually reproducing annelids includes ~24 putative species with two inferred origins of carnivory (Figure 1; Mack et al. 2023). Most species in this genus are small (< 5 mm in length) and putatively omnivorous. These small‐bodied forms, referred to here and previously as the *Chaetogaster* ‘*diastrophus*’ morphogroup, comprise the bulk of *Chaetogaster* diversity (including sp. 1, 2 and 5–21 in Figure 1) and are thought to subsist on a mixed diet of ciliates, detritus, algae and other tiny invertebrates like rotifers (McElhone 1980; Schonborn 1984; B. Streit 1977; Taylor 1980). The remaining species in the genus fall into two separate clades that specialise in consuming animal material. One, the *Chaetogaster* ‘*diaphanus*’ morphogroup, consists of two species (sp. 3 and sp. 4 in Figure 1) that are known to hunt invertebrates like crustaceans and other annelids (Green 1954; Mack et al. 2023; Monakov 1972). They possess an enlarged and muscular pharynx for suctioning prey and are the largest worms in the genus (~1 cm long). The other clade, the *Chaetogaster* ‘*limnaei*’ morphogroup, consists of three species (sp. 22, 23 and 24 in Figure 1) and is notable for obligate ecto‐ and endosymbiotic relationships with molluscs (Gruffydd 1965; Liquin et al. 2021; Smythe et al. 2015). Some lineages of *C*. ‘*limnaei*’ live on the surface of their host and are thought to consume small invertebrates, ciliates, algae and debris (B. Streit 1977), while other lineages specialise in consuming the kidney, ovary and gill cells of their host (Conn et al. 1996; Gruffydd 1965). Both the *C*. ‘*diaphanus*’ and *C*. ‘*limnaei*’ clades are bracketed by putatively omnivorous clades in the genus (Mack et al. 2023). Furthermore, most other species in the subfamily Naidinae are detritivores or algae‐feeders, including *Amphichaeta*, the sister genus to *Chaetogaster* (Erséus et al. 2017; Mastrantuono 1988). Thus, there is strong evidence indicating that carnivory is recently and repeatedly derived from noncarnivorous ancestors in *Chaetogaster*.

To infer how dietary shifts have occurred in *Chaetogaster*, a phylogenetic framework must be combined with dietary analyses across multiple *Chaetogaster* species. Recent multilocus phylogenies of *Chaetogaster* have uncovered cryptic diversity and achieved a deeper understanding of relationships between *Chaetogaster* species (Mack et al. 2023; Vivien et al. 2024). However, existing dietary reports for *Chaetogaster* are difficult to interpret. Many of these older studies identify gut contents solely based on morphology and are sourced from disparate habitats and localities. Moreover, studies reporting gut contents from many *Chaetogaster* species, including those on *C*. ‘*diaphanus*’, *C*. ‘*limnaei*’, *C*. ‘*diastrophus*’ and related species (e.g., *C*. ‘*langi*’), are unreliable, as these names are now understood to each refer to multiple different species, with potentially distinct diets (Mack et al. 2023; Vivien et al. 2024). As the diversity and relationships within the genus have come into focus, it is now feasible to investigate the dietary evolution of *Chaetogaster* through comparative gut‐content analyses.

A challenge in assessing the diet of wild animals is that gut processing can quickly render food items difficult or impossible to identify morphologically. Molecular techniques have emerged as powerful tools to identify otherwise unidentifiable gut contents across a range of organisms (Piñol et al. 2014). While host contamination is often present in these datasets, the sheer volume of reads retrieved from next generation sequencing platforms ensures that numerous sequences will be recovered from prey items. Recent studies have used this approach to characterise the diets of aquatic invertebrates and fish, using either universal cytochrome *c* oxidase subunit I (COI) primers to target animal prey (e.g., Casey et al. 2019; Eitzinger et al. 2019; Vasquez et al. 2021) or universal 18S primers to broadly detect eukaryotic prey (Albaina et al. 2016; Fernández‐Álvarez et al. 2018). The 18S gene is a particularly useful marker for broad investigations of eukaryotic gut contents in animals.

In this study, we use a metabarcoding approach to identify and compare gut contents across eight species of *Chaetogaster* and a detritivorous outgroup. We sequence 18S amplicons from field‐collected individuals and infer diets from the reads, complementing these data with visual assessments of gut contents. We then interpret these putative diets in the context of *Chaetogaster* phylogeny to gain insight into how carnivory evolved from noncarnivorous ancestors in the two carnivorous lineages. This study is novel both in the application of comparative metabarcoding to characterise diets across a freshwater invertebrate group and for the use of metabarcoding to understand the evolution of dietary strategies.

## 2 Materials and Methods

### 2.1 Specimen Collections and Imaging

Naidine annelids are patchily distributed, and little is known about their ecology. This makes targeted sampling of species infeasible. However, naidines are generally abundant between late spring and early fall in temperate freshwater habitats (McElhone 1978). To capture a diversity of *Chaetogaster* species, we broadly sampled in this period across 12 freshwater localities in Maryland and Pennsylvania, USA. Sampling involved the collection of sediment from lotic habitats and submerged aquatic vegetation from lentic habitats (to isolate *C.* ‘*diastrophus’* and *C. ‘diaphanus’* morphogroup worms) as well as snails from these same habitats (to isolate *C*. ‘*limnaei’* morphogroup worms).

In total, 117 worms were gathered across two collection efforts (Table S1). Between July and October 2020, 51 *Chaetogaster* worms were collected in the first sampling effort, hereafter referred to as Sampling 1. Between May and August 2021, another 55 *Chaetogaster* worms and 11 worms from the closely related detritivorous genus *Stylaria* were collected in a second sampling effort, hereafter Sampling 2.

After collection, field samples were stored in unaerated glass bowls with artificial spring water (ASpW) (1% artificial seawater made with InstantOcean salts) for 1–3 days at 16°C. Worms were recovered by swirling sediment or algal fronds in an Erlenmeyer flask, decanting through a 53 μm sieve, and sorting through the retained material using a stereomicroscope. To recover *C*. ‘*limnaei’* morphogroup worms, snails were crushed and dissected to extract worms. Live *Chaetogaster* and *Stylaria* specimens were cleaned by rinsing several times in ASpW and then imaged at 100X on an Axioplan2 microscope equipped with an AxioCam HRc camera (Zeiss, Oberkochen, Germany), documenting both the whole specimen and its gut contents. After imaging, specimens were preserved individually in 70%–80% ethanol and stored at 4°C. The time between isolation from the sample bowls and preservation in ethanol ranged from 1 to 3 h.

*Stylaria* worms were identified as one of two morphotypes (presumptive species) using Kathman and Brinkhurst (1998). Morphological identification of *Chaetogaster* worms was less feasible, as recent phylogenetic work indicates extensive cryptic diversity in the genus (Mack et al. 2023; Vivien et al. 2024). Using Kathman and Brinkhurst (1998), we initially classified *Chaetogaster* specimens as one of three morphogroups based on habitat, body size and morphology: the large generalist predator *Chaetogaster ‘diaphanus’* morphogroup, the small putative omnivore *Chaetogaster ‘diastrophus’* morphogroup and the mollusc symbiont *Chaetogaster ‘limnaei’* morphogroup. To refine our *Chaetogaster* identifications, we employed DNA barcoding with the COI locus to determine the identity of each of our specimens in reference to the putative species identified in Mack et al. (2023).

### 2.2 DNA Extraction and COI Barcoding

We extracted genomic DNA from each of the 117 specimens. Because these worms are small and their guts span their body length, the entire animal was used for extractions to capture potential prey DNA. Extractions were carried out using the DNAeasy Blood and Tissue kit (Qiagen), and DNA from each individual was eluted in 50 μL of TE buffer.

For barcoding of *Chaetogaster* specimens, polymerase chain reaction (PCR) was used to amplify a 709 bp fragment of COI with the primers LCO1490 (Folmer et al. 1994) and CHCr (Mack et al. 2023). The thermoprofile was 95°C (1 min); 35 cycles of 95°C (30 s), 45°C (1 min), 72°C (1 min); final extension at 72°C (5 min). Reactions (25 μL volumes) had a final concentration of 1X Buffer (Thermo Fisher Scientific), 2 mM MgCl_2_, 0.2 mM dNTPs, 0.4 μM of each primer, 1.25 units *Taq* Polymerase (Thermo Fisher Scientific) and 2 μL template DNA (or water for negative controls).

All COI PCR products were run on a 1.2% agarose gel to confirm amplification success and specificity, followed by spin‐column purification (MinElute PCR Purification Kit, Qiagen, Valencia, CA) and Sanger sequencing in both directions (Genewiz, Azenta Life Sciences). Forward and reverse reads were assembled in Geneious Prime (2020.0.0.5), and primer sequences were manually removed. All worms in the *C. ‘limnaei’* and *C. ‘diastrophus’* morphogroups were barcoded. Because prior phylogenetic work indicates that *C. ‘diaphanus’* worms collected across North America belong to a single species (Mack et al. 2023), we barcoded only a subset of *C. ‘diaphanus’* specimens collected in Sampling 2.

To assign specimens to numbered *Chaetogaster* species, we aligned their COI barcode sequences against the COI dataset used in Mack et al. (2023) with MAFFT online according to default settings (Katoh et al. 2019) and generated a neighbour‐joining tree using MEGA11 according to default settings (Tamura et al. 2021). Specimen barcodes were assigned to species if they nested within one of the clades identified as putative species in Mack et al. (2023).

### 2.3 18S Amplification and Sequencing for Metabarcoding

**FIGURE 2:**
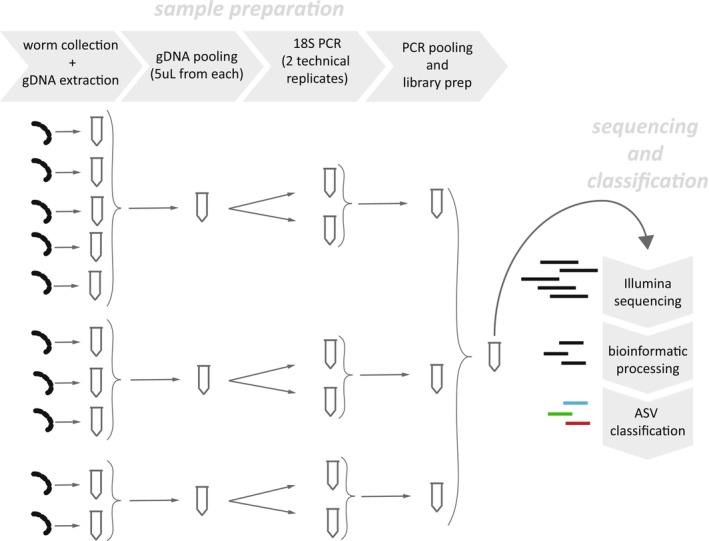
Metabarcoding pipeline. Illustration diagrams the main steps in the sampling and pooling protocol, from collection and DNA extraction to sequence classification. The illustration includes three hypothetical species samples with five, three and two worm specimens per species (from top to bottom).

We performed two metabarcoding sequencing runs, one for Sampling 1 (51 *Chaetogaster* worms) and another for Sampling 2 (55 *Chaetogaster* worms and 11 *Stylaria* worms). For each run, DNA extracts from individuals of the same species (*Chaetogaster*) or genus (*Stylaria*) were pooled together, followed by 18S PCR amplification and sequencing (Figure 2). The number of individual worms that contributed to each pooled sample ranged from 2 to 26 (Table S1). For each species of *Chaetogaster* represented in a sampling, we took 5 μL from each individual DNA extract and pooled these together to generate a single multi‐individual PCR template. For *Stylaria*, we did the same except that we combined 5 μL of DNA extracts from each *Stylaria* individual (whether *lacustris* or *fossularis* morphotype) to generate a single PCR template. *Stylaria* was pooled at the genus level because there remains considerable taxonomic uncertainty about the number of species in the genus, in addition to evidence for cryptic species (Brinkhurst 1986; Di Persia 1975; Horenkamp 2023). Furthermore, the two *Stylaria* morphotypes (*lacustris* and *fossularis*) are reported to occupy similar habitats, share a similar morphology, and are thought to have similar diets (Brinkhurst and Jamieson 1971; McElhone 1978; B. Streit 1978; Wachs 1967). Therefore, although pooling reduces resolution within *Stylaria*, the pooled sample represents a robust combined outgroup to *Chaetogaster*.

We used the primers Euk_B (Medlin et al. 1988) and 18S_V9_Con (O'Rorke et al. 2012), which amplify a 100–170 base pair segment of the 18S V9 region from a broad range of eukaryotes (Fernández‐Álvarez et al. 2018). For each pooled template, we ran two PCR reactions (technical replicates), each with a unique master mix. Reactions (25 μL) had a final concentration of 1X Buffer, 2 mM MgCl2, 0.2 mM dNTPs, 0.4 μM of each primer, 0.75 units of *Taq* polymerase and 2 μL template DNA (or water for negative controls). The thermoprofile was 95°C (5 min); 35 cycles of 94°C (30 s), 62°C (45 s), 72°C (1 min); final extension at 72°C (2 min). All PCR products were run on a 1.2% agarose gel to confirm amplification success and specificity, followed by spin‐column purification (Qiagen MinElute PCR Purification Kit).

Purified PCR products were quantified on a Hoefer DyNA Quant 200 Fluorometer, and the two replicates were pooled in equal DNA ratios for each species. We then generated libraries using the standard protocol for the NEBNext Ultra II DNA Library Prep Kit for Illumina (New England Biolabs), with 50 ng of input DNA. An Agilent 4150 TapeStation was used to quantify the libraries and verify quality. Six libraries were multiplexed and sequenced for the first sequencing run (Sampling 1), and eight libraries were multiplexed and sequenced for the second sequencing run (Sampling 2). Both runs were performed using paired‐end 2x150 bp sequencing on an Illumina MiSeq by Genewiz (Azenta Life Sciences) with a Phi‐X spike‐in (30% spike‐in for the first run, 20% spike‐in for the second run).

### 2.4 Illumina Read Processing and Taxonomic Assignments

The 18S sequences were de‐multiplexed and Illumina adapters were removed by Genewiz. Read processing and taxonomic identifications were accomplished using the DADA2 (v1.16) package in RStudio (Callahan et al. 2016). After trimming off primer sequences, removing reads with ambiguous nucleotides, merging forward and reverse sequences, and removing chimaeric sequences, a table of exact amplicon sequence variants (ASVs) was generated for each sample. Using this method, we were able to identify unique haplotypes in our sequencing dataset (Callahan et al. 2017). ASVs were then assigned to the lowest possible taxonomic ranking through alignment with a DADA2 formatted dataset of the SILVA (v132) 18S database (Morien and Parfrey 2018). The default 50% bootstrap confidence threshold was used for taxonomic assignment. This computationally intensive process was completed on the University of Maryland's former high‐performance computing cluster, Deepthought2. The R code used to process reads for Sampling 1 and Sampling 2 is provided in supplementary appendices S1 and S2, respectively.

Once ASVs were classified and outputted to tables, but prior to further calculations and plotting, we removed two groups of ASVs from the dataset. First, we removed all ASVs from annelids, as taxonomic resolution at the 18S V9 locus was insufficient to distinguish between annelid specimens and potential annelid prey. Second, we removed all ASVs from ‘dark taxa’, defined here as those that could not be identified beyond the level of Eukaryota.

We then used Microsoft Excel to calculate, for each of our samples, the relative proportions of all ASVs assigned to particular taxonomic groupings. We chose to represent animals at mostly the order and class level, but also included family and genus designations that were informative. Most of the ciliate (Ciliophora) sequences in our dataset were classified to the genus level, which enabled us to classify the reads as originating from free‐swimming (mobile) or anchored (sessile) groups, using family descriptions in Lynn (2010). Because the taxonomic resolution of diatoms, algae and fungi was poorer than that of the other groups, many such ASVs were only classified to family, order or class. We chose to group these taxa at higher taxonomic levels because members of these groups are likely to be similar in mobility and size, and thus of similar relevance to *Chaetogaster* feeding ecology. We then generated summary plots for each of our samples using Microsoft Excel, grouping ASVs into seven broad categories: Animals, mobile ciliates, sessile ciliates, diatoms, other algae, fungi and miscellaneous protists and potential host parasites. Plots were further edited for appearance using Adobe Illustrator.

## 3 Results

### 3.1 COI Barcoding of *Chaetogaster* Specimens

COI barcoding revealed that eight species of *Chaetogaster* were recovered from our sampling and that each of these could be assigned to a species previously identified in Mack et al. (2023). Barcodes for each of the *Chaetogaster* worms included in our study nested clearly within COI clades for either species 3, 8, 10, 12, 18, 20, 22 or 24 (Sampling 1: sp. 3, 8, 10, 12, 20, 22; Sampling 2: sp. 3, 8, 12, 18, 22, 24), following the species nomenclature of Mack et al. (2023). Many of the *Chaetogaster* worms in this study were collected from localities that were the same as or nearby those sampled in Mack et al. (2023), and each of our new COI sequences was identical or nearly identical to those previously reported in that study.

### 3.2 Sequencing Statistics

After removal of low‐quality sequences and chimaeras, the two sequencing runs produced a total of 11,568,803 reads (Sampling 1: 4,479,580; Sampling 2: 7,089,223; Table 1). Among these reads were a total of 1311 ASVs (Sampling 1: 65; Sampling 2: 1246; Table 1). As expected, most of the reads (11,195,790) and a few of the ASVs (80) were identified as coming from oligochaete annelids. These ASVs could not be reliably identified to the genus level, and we could not distinguish between annelid reads that originated from the host and annelid reads that might have originated from prey taxa. Because of this, all reads identified as annelid were discarded from the dataset.

**TABLE 1:** Summary of the reads and amplicon sequence variants recovered by metabarcoding. Shown are the total number of reads and amplicon sequence variants recovered for each *Chaetogaster* species and the *Stylaria* outgroup. Numbers represent combined totals from both Samplings 1 and 2.

Taxon	Total individuals included	Total reads recovered	Total ASVs recovered
*Chaetogaster* sp. 3	26	1,519,584	152
*Chaetogaster* sp. 8	23	1,890,314	409
*Chaetogaster* sp. 10	7	634,911	12
*Chaetogaster* sp. 12	17	1,971,692	378
*Chaetogaster* sp. 18	5	1,114,654	258
*Chaetogaster* sp. 20	17	1,367,124	23
*Chaetogaster* sp. 22	11	1,256,389	308
*Chaetogaster* sp. 24	2	1,241,223	276
*Stylaria*	12	572,912	404

The remaining 373,013 reads were identified as nonannelid. Of these nonannelid reads, 98,191 reads across 623 ASVs from both sequencing runs could not be identified beyond the level of ‘Eukaryota’ and were discarded as ‘dark taxa’. Following the removal of annelid and ‘dark taxa’ reads, our core analyses across all nine study taxa were based on a total of 274,822 reads and 608 ASVs.

### 3.3 Visual Assessment of Gut Contents

**FIGURE 3:**
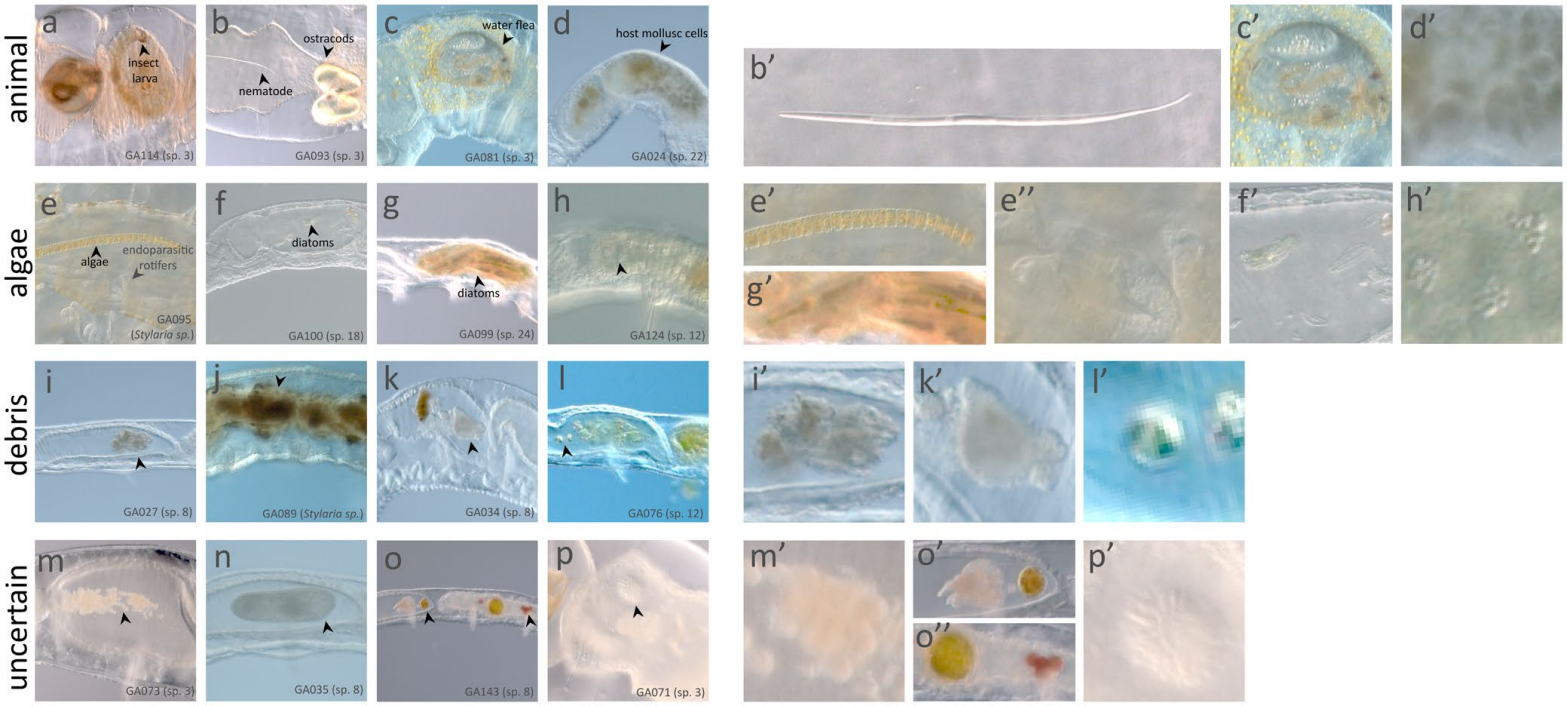
Representative images of *Chaetogaster* and *Stylaria* gut contents. Left panels show midgut regions of specimens, with some notable gut contents indicated by arrows. Right panels show a selection of gut contents from left panels, in a more magnified view. Anterior ends of *Chaetogaster* and *Stylaria* worms are oriented to the left. Specimen numbers and species are indicated in the lower right of the left panels.

Because naidine worms like *Chaetogaster* and *Stylaria* are largely transparent, some gut contents can be detected by inspecting whole worms under high magnification (Figure 3). To take advantage of this, we imaged all worms included in our molecular analyses and identified gut contents as well as possible. All specimens had material in their guts, but only some worms of each species had gut contents that could be easily identified. In the guts of *C*. sp. 3 (*C*. ‘*diaphanus*’ morphogroup), 42% (11/26) of worms had recognisable crustaceans in their guts and 4% (1/26) of worms had recognisable nematodes in their guts. Green material of algal origin was uncommon in the guts of *C*. sp. 3 worms, although in 19% (5/26) of worms, some photosynthetic organisms, especially globe algae (*Volvox* sp.) and diatoms, were observed. In a few cases, we observed these globe algae and diatoms passing through the gut intact. Meanwhile, the guts of *C*. ‘*limnaei*’ morphogroup worms were often found packed with cells that resembled (in cell size and pigmentation) those of their snail symbiont. These presumed mollusc cells were found in 55% (6/11) of *C*. sp. 22 worms and 100% (2/2) of *C*. sp. 24 worms. Algal cells were also observed in the guts of 9% (1/11) of *C*. sp. 22 individuals and 100% (2/2) of *C*. sp. 24 worms. For the five *C*. ‘*diastrophus*’ morphogroup species included in our dataset (*C*. sp. 8, 10, 12, 18 and 20), the identifiable gut contents varied considerably. In the guts of *C*. sp. 8 and *C*. sp. 10, brown debris and small individual cells made up most of the visible gut contents. It was difficult to determine which contents were detritus, animal, algal or single‐celled in origin. In *C*. sp. 12, 11% (2/17) of individuals had rotifers in their guts, and 30% (5/17) had algal cells in their guts. *Chaetogaster* sp. 18 individuals predominantly had diatoms in their guts, with 60% (3/5) of individuals having such contents. In *C*. sp. 20, brown debris could be discerned in the guts, but its origin was not clear.

In the guts of *Stylaria*, brown debris was typically found. Long filaments of algae were also observed in 20% (2/10) of worms. In 10% (1/10) of individuals, gut contents also included live rotifers, likely *Albertia* endoparasites (May 1989).

### 3.4 Taxonomic Categorisation of Amplicon Sequence Variants

A wide variety of eukaryotic groups were recovered from our 18S metabarcoding analyses (Table 2; Tables S2, S3, S4 and S5). Apart from a small number of ASVs that were likely contaminants (see below), most ASVs were considered as presumptive dietary items. We grouped ASVs together under seven categories of ecological relevance to *Chaetogaster* diets: animals, mobile ciliates, sessile ciliates, diatoms, other algae, fungi and miscellaneous protists/potential host parasites. This categorisation was done to better understand broad patterns in the evolution of *Chaetogaster* diets and make use of the ASVs which could not be identified to lower taxonomic levels.

**TABLE 2:** Summary of the major taxonomic groups recovered through metabarcoding analyses. Included are combined findings from both Samplings 1 and 2. Within each major category, taxa are listed in order of the read abundance of their representative sequences. Taxonomic groups represented by reads of relatively low abundance (< 1% of total reads per species) were omitted from this table.

Taxonomic category	Taxa recovered
Animals	Platyhelminth (Catenulida, Rhabdocoela, Digenea), water flea (Diplostraca), springtail (Collembola), snail (Heterobranchia), gastrotrich (Chaetonotida), sponge (Spongillida), ostracod (Podocopida), rotifer (Bdelloidea, Ploimida), cnidarian (*Craspedacusta*, Bivalvulida), nematode (Enoplida, Monhysterida), copepod (Cyclopoida), fairy shrimp (Sarsostraca)
Mobile ciliophorans	*Holosticha, Strobilidium, Telotrochidium, Cyclidium, Stylonychia, Stokesia, Homalogastra, Opisthonecta, Tachysoma, Urocentrum, Epalxella, Colpidium, Oxytrichia, Euplotes, Halteria, Caenomorpha*
Sessile ciliophorans	*Vorticella, Carchesium, Pseudovorticella, Trichodina, Epistylis, Trichodinella, Cothurnia*
Algae (diatoms and other)	Golden algae (Chrysophyceae, Chrysophyceae: Chromulinales, Ochromonadales, Synurales), diatoms (Bacillariophyceae, Mediophyceae), Dinoflagellata, green algae (Chlorophyceae), haptophytes (Prymnesiophyceae)
Fungi	Saccharomycetaceae, Trichomeriaceae, Aspergillaceae, Debaryomycetaceae, Chytridiomycetes, Didymellaceae, Leotiomycetes, Pleosporaceae, Sporidiobolaceae, Cryptomycota
Miscellaneous protists and/or potential host parasites	Perkinsidae, Eugregarinorida, Cercozoa, Echinamoebida, Heteronematina, Prokinetoplastina, Peronosporomycetes, Bicosoecida, Labyrinthulomycetes, Marine stramenopile clade 12, Neobodonida, Euglenaceae, Tetramitia, Flabellinia, Spumellaria, Entamoebida, Eubodonida
Potential contaminants and/or unlikely food items	Fungi (Malasseziaceae), arachnid (Opiliones, Acari), fish (Neopterygii), insect (Blattodea), mammal ( *Homo sapiens* )

Table 2 lists the groups represented by ASVs in each broad taxonomic category, organised by read abundance. The three most common ASVs for each major category were as follows: (i) animals: platyhelminths, arthropods, molluscs; (ii) mobile ciliophorans: *Holosticha* sp., *Strobilidium* sp., *Telotrochidium* sp.; (iii) sessile ciliophorans: *Vorticella* sp., *Carchesium* sp., *Pseudovorticella* sp.; (iv) diatoms: Bacillariophyceae; (v) other algae: Chrysophyceae and dinoflagellates; (vi) fungi: Saccharomycetaceae, Trichomeriaceae, Aspergillaceae and (vii) miscellaneous protist/potential host parasites: Perkinsidae, Eugregarinorida, Cercozoa.

In addition to the ASVs considered to be presumptive food items, we recovered a small number of reads that we deemed to be likely contaminants. In the sequencing run for Sampling 2, some ASVs for unexpected taxa were present in small amounts in all or most samples and were unlikely to represent dietary items. These included reads identified as trout, harvestmen, terrestrial mites, cockroach, human and *Malassezia* fungus. Collectively, they comprised 1.5% of total reads included in the analysis for Sampling 2. None of these ASVs were present in Sampling 1. These reads could have originated as lab contamination or were naturally present in the environment and amplified with PCR. They were not excluded from the analysis, but we characterised them as unlikely to be gut contents. It is possible that other reads in our dataset are environmental contaminants or otherwise unreflective of gut contents, but only these low‐abundance and widespread Sampling 2 reads were categorised as contaminants.

### 3.5 Inference of Gut Contents From Metabarcoding Data

**FIGURE 4:**
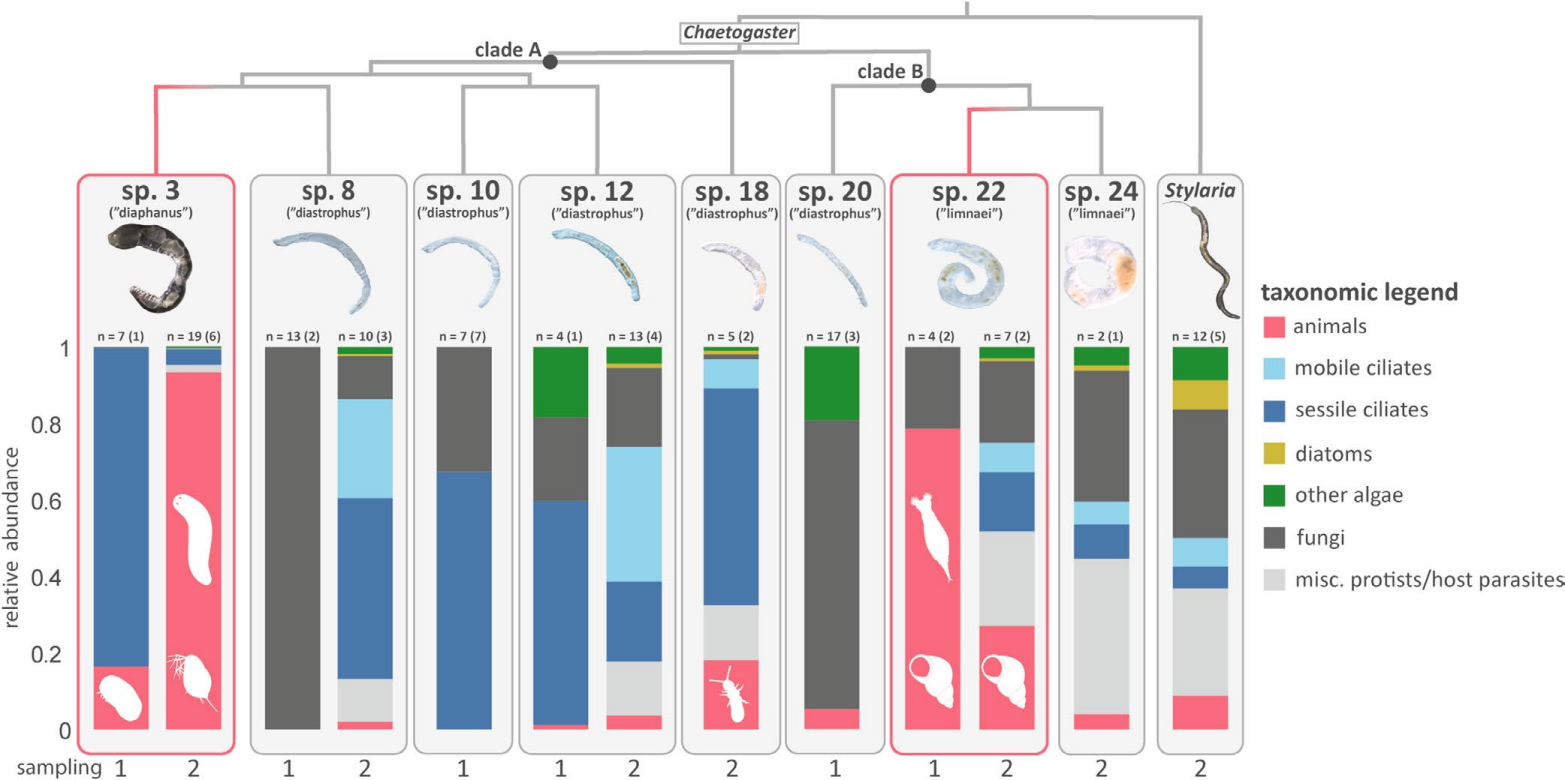
Taxonomic composition of 18S metabarcoding reads. Shown are the proportion of eukaryotic 18S V9 reads recovered from each species and sampling. Taxonomic assignments at multiple levels were collapsed into seven broad categories. Reads that were unclassified beyond the level of Eukaryota or identified as annelid were removed. Organism silhouettes over the bars represent some of the dominant animal taxa recovered. Above each sample plot, the number of worm specimens included in that sample is shown, followed by the number of collection sites represented in the sample, in parentheses. The phylogenetic backbone is adapted from Mack et al. (2023). Clades A and B represent the monophyletic groupings of the *C. ‘diaphanus’* and the *C. ‘limnaei’* morphogroups with their closest relatives respectively.

The taxonomic composition of 18S reads varied across *Chaetogaster* species and *Stylaria*, indicating substantial dietary differences among our study species (Figure 4, Table S6). Each species yielded a diversity of presumptive food items, consistent with each taxon having a mixed diet. In general, metabarcoding profiles from Sampling 2 had greater diversity than Sampling 1, though species that were included in both samplings showed some consistent patterns. Below, we provide overviews of the metabarcoding results for each study taxon. Throughout, when describing the proportion of reads classified as being from a particular taxonomic group, we report this as a percentage of the total reads from that annelid species (or genus, for *Stylaria*). The total used in these calculations includes the small number of reads from suspected contaminants but excludes reads identified as annelid as well as unidentifiable (‘dark taxa’) reads.

The pooled *Stylaria* sample ( *S. lacustris* and *S. fossularis* ) was dominated by reads from fungi (34%), miscellaneous protists/host parasites (28%) and photosynthetic eukaryotes (diatoms and other algae) (17%). Of all the study species, the *Stylaria* sample had the greatest proportion of diatom reads (8%). Although about 9% of the total reads were from animals, almost all of these were from the presumed contaminants present in Sampling 2. Reads from noncontaminant aquatic animals collectively represented only 1% of the total number from *Stylaria*. These originated from diverse taxa, including rotifers, platyhelminths, nematodes, gastropods, copepods, fish and gastrotrichs, most of which are deemed unlikely food items. Reads from ciliates represented 14% of total reads, with mobile ciliates representing 8% and sessile ciliates representing 6%. These ciliate fractions were among the lowest in our dataset.

The eight *Chaetogaster* species included in this study fall into two clades, which we refer to as Clade A and Clade B. Each includes a carnivorous lineage. From Clade A, our study included four species from the small, putatively omnivorous *C. ‘diastrophus’* morphogroup (*C*. sp. 8, 10, 12 and 18) and one species from the generalist predator *C. ‘diaphanus’* morphogroup (*C*. sp. 3). From Clade B, our study included one species from the small, putatively omnivorous *C. ‘diastrophus’* morphogroup (*C*. sp. 20) and two species from the mollusc‐associating *‘C. limnaei’* morphogroup (*C*. sp. 22 and 24).

Within *Chaetogaster* clade A, a striking result is that all five species showed a large fraction of ciliates in their metabarcoding profiles (for at least one of the samplings), in contrast to the profiles of species in clade B and the *Stylaria* sample. Among the *C. ‘diastrophus’* morphogroup species, one sample (*C*. sp. 8 in Sampling 1) had no detected ciliates, but in most of the other samples (including Sampling 2 for *C*. sp. 8), the fraction of ciliates (mobile and sessile combined) was consistently high, ranging from 56% to 83%. In *C*. sp. 3 (*‘diaphanus’*), that ciliate fraction was only 4% in Sampling 2 but was 83% in Sampling 1. Across Clade A species, the sessile ciliate *Vorticella* was in particularly high read abundance and was often the most abundant or sole member of the sessile ciliate fraction.

Beyond this large ciliate representation in Clade A, the metabarcoding profiles of the four *C. ‘diastrophus’* morphogroup species (*C*. sp. 8, 10, 12 and 18) differed substantially from that of the *C. ‘diaphanus’* morphogroup species (*C*. sp. 3). Among the *C*. ‘*diastrophus’* species, ciliates and/or fungi collectively made up most reads, ranging from 66% to 100%. For *C*. sp. 8, the closest relative of the carnivorous *C*. sp. 3, results from the two samplings differed, with the Sampling 1 profile comprising exclusively *Penicillium* fungi (Aspergillaceae), and the Sampling 2 profile being mixed, including 47% sessile (primarily *Vorticella*) ciliates, 26% mobile ciliates, 11% fungi and 2% algae. The animal fraction was small, only 2%, of which 0.2% was classified as sponge. *Chaetogaster* sp. 10 had 67% of reads being sessile ciliates (exclusively *Vorticella*) and 33% of reads being fungi (mostly *Penicillium*). *Chaetogaster* sp. 12 had ciliates making up the largest portion of the reads in both samplings, with Sampling 1 yielding 59% sessile ciliate reads (mostly *Vorticella*) and Sampling 2 yielding 35% mobile ciliate reads plus 21% sessile ciliate reads. Fungi made up the next most abundant fraction, 21%–22%. Only a very small fraction of reads were derived from animals (1% in Sampling 1% and 4% in Sampling 2), originating from a range of animal groups (water flea, sponge, copepod, platyhelminth, rotifer and gastrotrich). Finally, for *C*. sp. 18, the metabarcoding profile was dominated by ciliates, with 57% from sessile ciliates (entirely *Vorticella*) and 8% from mobile ciliates. The animal fraction in this species was 18%, the second largest animal fraction within clade A after *C*. sp. 3. These reads consisted primarily of springtail (10%) and gastrotrich (6%), with a small fraction of reads also from other taxa (rotifer, sponge, copepod, myxosporean and platyhelminth, each < 0.3%).

In comparison to these *C. ‘diastrophus’* morphogroup species within Clade A, the metabarcoding profile of the known predator *C*. sp. 3 (*‘diaphanus’* morphogroup) is sharply contrasted. Its dietary profile consisted almost exclusively of reads from animals and sessile ciliates. In Sampling 1, the dietary profile was dominated by *Vorticella* reads (83%), plus a significant number of reads in the animal fraction (16%), all of which were from ostracods. In Sampling 2, the dietary profile was instead dominated by animal reads (93%), this time composed of 65% from free‐living platyhelminths, 23% from diplostracans (water fleas) and 0.04% from nematodes. Reads from sessile ciliates were also recovered in this sampling, albeit at a lower abundance of 4%, all of which were from *Vorticella*. In both Sampling 1 and 2, this species had a very small number of fungal reads and of algal reads (each < 1%).

Within *Chaetogaster* clade B, the three species (one in the ‘*diastrophus’* morphogroup and two in the mollusc‐associating ‘*limnaei*’ morphogroup) had in common a relatively small or absent fraction of ciliate reads and a modest to large fraction of fungal reads. Beyond that, the species differed substantially from each other in their metabarcoding profiles. *Chaetogaster* sp. 20 (*‘diastrophus’* morphogroup) produced a metabarcoding profile with a large proportion of fungal reads (76%) and a modest proportion of nondiatom algal reads (20%), all of which were from the mixotrophic flagellate *Poterioochromonas* (Chromulinales). Its profile also had a small animal fraction, only 5%, which was comprised of rotifer reads. *Chaetogaster* sp. 24, one of the two *C. ‘limnaei’* morphogroup species in our dataset, yielded a dietary profile that was remarkably similar to that of *Stylaria*. This species had relatively large fractions of miscellaneous protist/potential host parasite reads (41%) and fungal reads (34%), as well as some ciliate reads (15%) and algae and diatoms (6%). This *Chaetogaster* species yielded a small fraction of animal sequences (4%), composed of platyhelminth, sponge and rotifer (each < 1%). Although this species is a snail symbiont and all specimens were collected from snails, no gastropod reads were detected. *Chaetogaster* sp. 22, the second mollusc‐associating *C. ‘limnaei’* morphogroup species, had a much greater proportion of animal reads than *C*. sp. 24 but also showed similarities to the mixed dietary profile of *C*. sp. 24 and *Stylaria*. For Sampling 1, the metabarcoding profile of *C*. sp. 22 had an animal fraction of 80%, nearly as high as that of *C*. sp. 3 (Sampling 2). These animal reads originated predominantly from gastropod mollusc (70%), with a smaller fraction coming from rotifer (8%). The remainder was almost entirely fungal reads identified as *Penicillium* (21%). For Sampling 2, the metabarcoding profile had a lower but still substantial animal fraction, 27%, with 11% originating from gastropod mollusc. Beyond the animal fraction, this species had a mixed dietary profile similar to *C*. sp. 24 and *Stylaria*, with miscellaneous protist/potential host parasite (25%, dominated by Perkinsidae), ciliates (23%), fungi (21%) and a small fraction of reads from diatoms and other algal groups.

## 4 Discussion

Little is known about how or why carnivory evolves in animals, in part because recent and repeated origins of carnivory are sparse (Burin et al. 2016; Price et al. 2012; Román‐Palacios et al. 2019; Siqueira et al. 2020). To understand the evolution of carnivory, it is important to infer the dietary steps that might link closely related noncarnivorous and carnivorous taxa by studying groups that include both noncarnivorous and carnivorous species. Across such groups, general information on diets can be inferred from morphological adaptations and behaviour, but specific information about the identity of ingested items is invaluable. Here, we performed a study of the diets of eight closely related species of freshwater annelids and a close outgroup, employing both metabarcoding and visual gut content analyses to characterise the diets of presumed carnivorous and noncarnivorous species collected from the wild. We then used these data to infer key steps in the evolution of carnivory within this group of annelids.

Metabarcoding of small aquatic invertebrates is a difficult endeavour, especially when working with a genus like *Chaetogaster* that contains cryptic diversity (Mack et al. 2023; Vivien et al. 2024). Our study involved sampling tiny *Chaetogaster* worms that are patchily distributed and could not be identified until after they were barcoded. We then used metabarcoding to study the (mostly) even tinier organisms that comprised their gut contents. The necessarily opportunistic sampling led to varied representation of target species, and taxonomic identification of putative gut contents remains somewhat coarse. Despite these challenges, the dual origins of carnivory within *Chaetogaster* provided a rare and powerful opportunity to investigate evolutionary transitions to carnivory.

### 4.1 New Evidence Supporting Two Origins of Carnivory in *Chaetogaster*

Prior knowledge suggested that there were both carnivorous and noncarnivorous lineages in *Chaetogaster*, but it was unclear how many of the lineages showed diets composed primarily of animals, nonanimals or a mixture of both. Nor was it clear how many times carnivorous diets evolved in the genus. With a molecular and visual gut content analysis across species, we confirm that carnivory evolved twice in *Chaetogaster* from noncarnivorous, likely detritivorous ancestors. Members of both the *Chaetogaster ‘diaphanus’* (*C*. sp. 3) and *Chaetogaster ‘limnaei’* (*C*. sp. 22) clades show large proportions of 18S reads from freshwater invertebrates that are consistent as prey items. These *Chaetogaster* species also have a strong representation of animal tissue in visually detectable gut contents. Together, these findings solidify the conclusion that these species are carnivorous. These results are in stark contrast to those for *Stylaria* and the other *Chaetogaster* species in our dataset, all of which have metabarcoding profiles that are dominated by algae, ciliates or fungi. High representation of algae and ciliates is best interpreted as being indicative of grazing on periphyton or ingesting colonised sediments, whereas large proportions of fungal reads are likely indicative of detritivory, as freshwater detritivores favour plant debris that is already colonised by fungi (Bärlocher 1985; Graça et al. 1993).

Species 3 of the *C. ‘diaphanus’* clade appears to show the diet most reliant on animals in our dataset. In Sampling 2 especially, most of the 18S reads recovered for this species were identified as animals, primarily arthropods and platyhelminths. Prior dietary analyses only reported hard‐bodied organisms like ostracods, water fleas and nematodes as food for *C. ‘diaphanus’* (Green 1954; Monakov 1972). Platyhelminths, which dissolve quickly in the gut and leave no hard structures, are a newly reported dietary item for *C*. sp. 3 worms. Although platyhelminth reads could theoretically be derived from parasites within the *C*. sp. 3 individuals sampled, we found that all the platyhelminth reads associated with *C*. sp. 3 were identified as Rhabdocoela or Catenulida, which are predominantly free‐living groups (Kolasa 1991). Thus, we conclude that arthropods, nematodes and platyhelminths all represent significant prey items for *C*. sp. 3. A diet composed of such mobile prey is consistent with the observed behaviour of *C*. sp. 3 in laboratory cultures, where worms are known to roam in search of food and ingest small invertebrates. In the wild, it is likely that *C*. sp. 3 worms also roam to maximise the probability of encountering and ambushing other animals.

The second species to show a large fraction of animal tissue is *C*. sp. 22 of the mollusc‐associating *C. ‘limnaei’* clade. This species had up to 76% animal reads in its metabarcoding profile, the largest fraction of which was gastropod in origin. In the past, researchers have recognised an ecological dichotomy within *C. ‘limnaei’*: one subgroup (originally considered a subspecies) was described as exclusively endosymbiotic on molluscs, feeding on cells from the host's kidney, ovaries or gills, while another was described as exclusively ectosymbiotic on molluscs, living on snails but feeding on a mixed diet of debris, algae and small invertebrates (Gruffydd 1965; B. Streit 1977). Phylogenetic analyses of *Chaetogaster* have since complicated this view of *C. ‘limnaei’*. The group is now recognised to include at least three species (Mack et al. 2023; Smythe et al. 2015), two of which were represented in our dataset. Our sampling of *C. ‘limnaei’* morphogroup individuals included specimens found both exteriorly and interiorly in snails, and although we cannot make claims about where on the snail each species tended to be found, both our image‐based and metabarcoding results clearly show that only *C*. sp. 22 has a strongly carnivorous diet of host tissue and rotifers. By contrast, *C*. sp. 24 displays a dietary profile indicative of detritivory and/or periphyton grazing, with only a small animal fraction but a large fraction of reads from fungi. We also recover a smaller but still substantial fraction of ciliates in this species. Algal consumption was also confirmed through visual assessment of gut contents. The general dietary profile observed in *C*. sp. 24 was like that of other nonpredatory *Chaetogaster* species in our dataset as well as the *Stylaria* outgroup, suggesting that the diet of *C*. sp. 24 might be similar to the ancestral diet for this group. Overall, our findings suggest that *C*. sp. 24 is primarily ectosymbiotic and nonparasitic (as proposed in Mack et al. (2023)), while *C*. sp. 22 may be endosymbiotic and parasitic (or at least host‐cell consuming). Future work on these two species will be needed to further test these characterisations, as well as to establish the diet of the third C. ‘*limnaei’* morphogroup species that was not recovered for this dataset.

### 4.2 Caveats Related to the Interpretation of Metabarcoding Data

Metabarcoding has emerged as a prominent tool for the assessment of diets (Alberdi et al. 2019; Piñol et al. 2014). It is especially useful for tiny organisms like *Chaetogaster*, where a visual identification of even tinier food items is particularly challenging (e.g., Fernández‐Álvarez et al. 2018; Flo et al. 2024; Vasquez et al. 2021). However, there are a few caveats in all metabarcoding analyses that must be considered, including in our study.

First and foremost are limitations related to the taxonomic identification of reads. Approximately one quarter of the (nonannelid) reads in our dataset could not be identified beyond the level of Eukaryota and were thus excluded as ‘dark taxa’. Some of these ‘dark taxa’ likely represented real dietary items for *Chaetogaster* or *Stylaria* worms, but remained effectively undetected because they could not be identified with sufficient resolution. Incomplete reference databases with taxonomic and geographical biases (Keck et al. 2023; Porter and Hajibabaei 2018; Weigand et al. 2019), the use of different molecular markers across studies (Casey et al. 2021; Macheriotou et al. 2019) and the performance of bioinformatic tools (Deiner et al. 2017; Hleap et al. 2021; Somervuo et al. 2017) can all lead to unidentifiable ‘dark taxa’ in environmental metabarcoding and metagenomic studies (Casey et al. 2021; DiBattista et al. 2020; Ransome et al. 2017). Despite these limitations, we were able to identify the large majority of reads in our study at least to kingdom and were able to identify patterns in our data. However, future metabarcoding studies will benefit from initiatives to improve the taxonomic coverage of reference databases and the performance of classifiers.

We also recognise that our pooling of individual genomic DNA extracts prior to amplification obscured any potential intraspecific variation in dietary profile. For example, *Chaetogaster* worms may show differences in diet according to microhabitat, size or age. While identifying such variation was not the goal of the current study, it would be fruitful in future work to sequence individual worms to investigate individual‐level dietary profiles and potential patterns.

Within our pooled samples, it is also important to recognise that read abundances are unlikely to be strictly correlated with dietary composition. Amplification biases, differential DNA decay rates and variation in molecular marker copy number across potential dietary items can all affect the relationship between dietary composition and read abundance. For example, in our 18S dataset, there is a potential for a bias towards an overrepresentation of ciliate reads. This is because peritrichs like *Vorticella* can have over 300,000 copies of 18S per cell (Gong et al. 2013). In contrast, fungi, animals or other protists may only have hundreds to tens of thousands of 18S copies (Prokopowich et al. 2003; Simon et al. 2005). This difference could lead to an overrepresentation of ciliate reads compared to other gut contents that is not reflective of actual dietary preferences. However, it is likely that the relative proportions of ciliate reads that we recovered are at least somewhat representative of actual worm diets. Even if ciliate reads tend to overrepresent the relative ciliate fraction in the diet, our metabarcoding data indicate substantial differences in the ciliate fraction between taxa, including consistent differences between species in Clade A and Clade B. Furthermore, a preference for ciliates among some *Chaetogaster* species has been noted in the past (Schonborn 1984; Taylor 1980), and we see evidence in many of our gut content images of organisms that could be single‐celled eukaryotic prey items. It is also important to acknowledge that some reads recovered in our dataset could come from the external environment as eDNA or may exist as organisms that live on or inside the food items that are eaten by the worms. Despite this potential caveat, there are a few reasons why we can still have confidence in the observed patterns. First, we can corroborate the taxa that are present in the metabarcoding dataset with visual data. Algal cells, detritus and arthropod prey items are all present in both the 18S data and gut content images of individuals in our dataset. Second, we also have species pairs, such as *Stylaria* and *C*. sp. 3, that were collected from overlapping localities yet show distinct molecular dietary profiles. Compared to gut content DNA, the contribution of reads from the external environment is likely to be low.

Finally, the potential for temporal variation in diet is also important to consider. In our dataset, we noticed considerable differences between the dietary profiles for some species that were collected in both Samplings 1 and 2. While these could be attributed to stochastic events during PCR or sequencing, they may also be a consequence of the time when each species was sampled across our two sampling efforts, thus potentially reflecting seasonal variation or year‐to‐year variation. Previous work indicates that the diets of freshwater invertebrates and even entire trophic webs can change seasonally, as nutrient inputs and community composition fluctuate (Miyasaka and Genkai‐Kato 2009; Schmid‐Araya et al. 2016). In particular, spring and early summer are a time when freshwater communities are known to be more diverse as dissolved oxygen levels are higher (Croijmans et al. 2021). It is, therefore, possible that the greater biodiversity of this part of the year could explain why Sampling 2 yielded substantially more reads and amplicon sequence variants than did Sampling 1. Future work that rigorously investigates seasonal and inter‐year variation of molecular dietary profiles for *Chaetogaster* and other meiofaunal organisms would be worthwhile, as these animals are likely to have dynamic diets that cannot be captured in a single snapshot or sampling period.

### 4.3 *Evidence for a Ciliate‐Mediated Transition to Carnivory in the Chaetogaster* ‘diaphanus’ *Clade*

The *C. ‘diaphanus’* morphogroup, which includes *C*. sp. 3, has long been recognised as morphologically and behaviourally unusual worms. They comprise large, active hunters, with an increased body size (0.5–2 cm long) and muscular heads. Their morphology distinguishes them from every other *Chaetogaster* species, which are typically tiny (1–2 mm long), less muscular and morphologically alike (Mack et al. 2023; Sperber 1948). Our study provides the first data available to infer the dietary transitions that led to this highly unusual predatory annelid lineage.

We find general support for the hypothesis of a gradual shift towards more mobile prey during the evolution of the *C*. sp. 3 lineage. Across *Chaetogaster* clade A, all species sampled have relatively large proportions of reads originating from ciliates, whereas species in Clade B and the *Stylaria* outgroup show much lower ciliate read counts. This suggests that there was a transition at the base of Clade A towards much greater consumption of ciliates. Ciliates as a group are among the fastest moving unicellular eukaryotes, and even the sessile forms include species with very fast reactions (e.g., stalk contractions) (Lisicki et al. 2019; Ryu et al. 2012). This dietary transition at the base of Clade A thus represents a shift to faster moving food items. A transition towards strong predation specifically on animals likely appeared closer to the origin of *C*. sp. *3*, as other Clade A species have relatively few animal reads. Instead, these other Clade A species (*C*. sp. 8, 10, 12 and 18) show dietary profiles that are mostly dominated by fungi and/or ciliates. Together, these data indicate that generalist carnivory in the *C. ‘diaphanus’* clade may have evolved from detritivorous ancestors that increasingly specialised first on ciliates, rather than directly on other animals. Furthermore, the closest relative in our dataset to the *C. ‘diaphanus’* clade, *C*. sp. 8, has a relatively large fraction of ciliate genera classified as mobile. This finding suggests the possibility that adaptations for feeding on ciliates that are mobile (rather than sessile) might have been an additional step towards the evolution of predation on mobile invertebrate prey in the ancestors of the *C. ‘diaphanus’* clade.

Overall, our data suggest that generalist carnivory can evolve from noncarnivory through gradual dietary shifts involving increasingly active prey. Organisms adapted to feeding on non‐ or slow‐moving food items (e.g., detritus, algae) may find a pathway to carnivory through multiple steps, including (1) adaptations for feeding on attached but fast‐reacting food items (e.g., sessile ciliates), followed by (2) adaptations for feeding on faster‐moving and freely moving food items (e.g., mobile ciliates) and finally (3) adaptations for feeding on increasingly larger mobile prey, most of which are likely represented by larger mobile animals.

### 4.4 Evidence for a Symbiosis‐Mediated Transition to Carnivory in the *Chaetogaster* ‘*limnaei’* Clade

Among naidine annelids, just as remarkable as the voracious carnivory of the *C. ‘diaphanus’* clade are the mollusc associations of the *C. ‘limnaei’* clade. Compared to most clitellates, which are largely free‐living, worms in the *C. ‘limnaei’* clade are unusual in that they appear to have an obligate symbiosis with gastropod molluscs. Some lineages are thought to be merely ectosymbiotic, but others are presumed to be parasitic (Conn et al. 1996; Gruffydd 1965; Smythe et al. 2015; B. Streit 1977). The close association of *C. ‘limnaei’* worms with molluscs suggests that this clade may have transitioned to carnivory through feeding on host tissues, rather than the ciliate‐mediated transition that we hypothesise for the *C. ‘diaphanus’* clade. If a symbiosis‐mediated transition to carnivory occurred during the evolution of *C. ‘limnaei’*, we would expect to find that mollusc symbiosis precedes the evolution of carnivory, and we would not expect to find an increase in the proportion of ciliate reads in the nearest relatives of the carnivorous *C. ‘limnaei’* lineage.

Our dataset supports both expectations. The one free‐living species in Clade B, *C*. sp. 20, shows a dietary profile indicative of detritivory, with metabarcoding reads composed almost entirely of fungi and algae, and only amorphous material visible in the imaged guts. *Chaetogaster* sp. 22 and 24, the two representatives of the mollusc‐associating *C. ‘limnaei’* clade in our dataset, differ substantially in dietary profiles. The latter has a molecular dietary profile that is almost entirely nonanimal and is remarkably similar to that of the detritivorous outgroup *Stylaria*. Although material that resembled snail tissue was visually detected in the guts of *C*. sp. 24 specimens, no mollusc reads were detected for this species. However, it is important to recognise that only two individuals of *C*. sp. 24 were recovered in this study, limiting our preliminary inference of diet. Sampling of additional *C*. sp. 24 individuals may reveal the presence of mollusc and/or other dietary items not detected in this analysis. In contrast to *C*. sp. 24, the dietary profile of *C*. sp. 22, of which 11 individuals were recovered from multiple sites, shows a large proportion of animal reads (especially so in Sampling 2). This pattern indicates a significant reliance on animal tissue for food. Ciliates are present in some clade B species, but in none are they a large fraction of the dietary profile. Together, these data support a symbiosis‐mediated transition to carnivory in the *C. ‘limnaei’* clade, as mollusc association is inferred to have evolved prior to the evolution of substantial feeding on the mollusc host. Nonetheless, our dataset provides no evidence for intermediate diets between detritivory and carnivory in Clade B, suggesting that this symbiosis‐mediated transition to carnivory may have been more rapid than the gradual steps towards generalised carnivory in clade A.

Symbiotic interactions have been implicated in the evolution of carnivory and parasitism in other invertebrate groups as well. For example, lepidopteran species with myrmecophagous caterpillars tend to be descended from clades that form commensal associations with ants (Als et al. 2004; Pierce 1995). Similarly, initial stages of commensal ecto‐ and endosymbiosis have been proposed as pathways from free‐living to parasitic strategies in umagillid platyhelminths and *Strongyloides* nematodes (Jennings 1997; Shinn 1981; A. Streit 2014). An endosymbiotic association may have also preceded the evolution of parasitism in the annelid naidine genus *Dero*, some representatives of which parasitise frog ureters (Andrews et al. 2015). *Dero* and *Chaetogaster* are relatively closely related, being within the same subfamily, and are the only confirmed transitions to endoparasitism among clitellate oligochaetes, making them promising models for future comparative studies on how this unusual trophic strategy might evolve.

### 4.5 *Chaetogaster* as an Important Component of Freshwater Food Webs

Meiofaunal invertebrates have historically been viewed as a homogenous assemblage of primary consumers with a limited impact on ecosystems, but evidence is mounting that they have significant roles in freshwater systems (Peralta‐Maraver et al. 2023). Despite their small size, they are incredibly abundant and many meiofaunal species are now recognised to occupy relatively high positions in trophic networks as secondary consumers (Palmer 1990; Schmid‐Araya et al. 2016). Our data further contribute to this awareness by demonstrating that many species of *Chaetogaster*, including even the smallest representatives (1–2 mm long), consume not just detritus and algae but also large numbers of ciliates and other invertebrates. The ecological significance of symbiotic worms in the *C. ‘limnaei’* clade has long been recognised (Michelson 1964; Stoll et al. 2013), but our study indicates that the generalist predators in the *C. ‘diaphanus’* clade and their smaller omnivorous relatives could also be significant in freshwater food webs as both primary and secondary consumers.

### 4.6 The Evolution of Carnivory in Annelids

Annelids display a great diversity of feeding modes. Although detritivory, periphyton grazing, scavenging and omnivory are particularly common, transitions to carnivory have occurred repeatedly across the phylum. Among Errantia, for example, voracious predators are known from Eunicidae, Nereidae and Onuphidae (Jumars et al. 2015), and among Sedentaria, predatory and/or parasitic species are known among Naididae (*Chaetogaster*, *Dero*), Lumbriculidae (*Phagodrilus*, *Agriodrilus*) and the largest clade of annelid predators, the Hirudinea (leeches and relatives) (Brinkhurst and Jamieson 1971; McKey‐Fender and Fender 1988). Carnivorous annelid lineage origins range from very recent (e.g., *Chaetogaster*) to ancient (e.g., leeches) (Erséus et al. 2017; de Carle et al. 2025). Annelids are thus a useful clade for identifying broad patterns in the evolution of carnivory. One lesson from *Chaetogaster* is that transitions to carnivory can take different paths, even among close relatives. In *Chaetogaster*, we found evidence for one transition to carnivory mediated by feeding on increasingly mobile food items and another mediated by symbiosis with another animal. There is evidence that a symbiosis‐mediated transition to carnivory may be a recurrent pathway in annelids. The leeches and acanthobdellidans, a clade of carnivores and parasites, are most closely related to crayfish ectosymbionts (branchiobdellidans) (Tessler et al. 2018). This phylogenetic pattern is consistent with a symbiosis‐mediated path to carnivory from detritivory. Most other carnivorous annelid lineages, however, stem from clades without known animal symbioses. A pathway to carnivory via feeding on increasingly mobile food sources, as indicated for the *C. ‘diaphanus’* clade, is thus an important model to be tested in these other lineages, using the comparative metabarcoding approaches employed here. More broadly, further dietary studies on annelids and other animal groups will be important to assess whether the two pathways recovered in *Chaetogaster* represent the primary recurrent pathways to carnivory, or whether additional pathways exist for these extraordinary trophic transitions.

## Author Contributions

J.M.M. and A.E.B. conceived the study and planned its design. J.M.M. did the field sampling and collected and analysed the data. J.M.M. wrote the original draft of the manuscript. Both J.M.M. and A.E.B. edited the manuscript and figures.

## Conflicts of Interest

The authors declare no conflicts of interest.

## Supporting information


**Appendix S1:** Supporting Information

**Appendix S2:** Supporting Information

**Table S1:** Collection information for specimens included in this study.

**Table S2:** Output table from DADA2 listing the taxonomic identification of each amplicon sequence variant (ASV) and its corresponding 18S sequence for Sampling 1. ASVs labelled as NA in the accession number column could not be identified to a specific sequence in the database and had multiple ambiguous matches.

**Table S3:** Output table from DADA2 listing the number of reads per each amplicon sequence variant (ASV) recovered from the species included in Sampling 1.

**Table S4:** Output table from DADA2 listing the taxonomic identification of each amplicon sequence variant (ASV) and its corresponding 18S sequence for Sampling 2. ASVs labelled as NA in the accession number column could not be identified to a specific sequence in the database and had multiple ambiguous matches.

**Table S5:** Output table from DADA2 listing the number of reads per each amplicon sequence variant (ASV) recovered from the species included in Sampling 2.

**Table S6:** Relative proportions of amplicon sequence variant (ASV) counts across the broad taxonomic categories for each species across both samplings.

**Appendix S3:** Supporting Information

## Data Availability

Raw 18S rDNA reads are available on the NCBI Sequence Read Archive (PRJNA1337925). Amplicon sequence variant (ASV) sequences, taxonomic identifications, and read counts, in addition to collection locality information, can be found in the supplementary files.
