# Supplementary material for: From Mud to Meat: Comparative Metabarcoding Reveals Two Different Evolutionary Paths to Carnivory in a Group of Meiofaunal Annelids
Source: Mol Ecol. 2025 Oct 28;34(22):e70151. doi: 10.1111/mec.70151 (PMC12617034; doi:10.1111/mec.70151)
Supplement: Supplementary file 9 — Appendix S3: Supporting Information [file MEC-34-e70151-s003.docx]

**Supplementary table 1.** Collection information for specimens included in this study.

**Supplementary table 2.**  Output table from DADA2 listing the taxonomic identification of each amplicon sequence variant (ASV) and its corresponding 18S sequence for Sampling 1. ASVs labelled as NA in the accession number column could not be identified to a specific sequence in the database and had multiple ambiguous matches.

**Supplementary table 3.** Output table from DADA2 listing the number of reads per each amplicon sequence variant (ASV) recovered from the species included in Sampling 1.

**Supplementary table 4.** Output table from DADA2 listing the taxonomic identification of each amplicon sequence variant (ASV) and its corresponding 18S sequence for Sampling 2. ASVs labelled as NA in the accession number column could not be identified to a specific sequence in the database and had multiple ambiguous matches.

**Supplementary table 5.** Output table from DADA2 listing the number of reads per each amplicon sequence variant (ASV) recovered from the species included in Sampling 2.

**Supplementary table 6.** Relative proportions of amplicon sequence variant (ASV) counts across the broad taxonomic categories for each species across both samplings.
